# Malignant mesothelioma in the State of Qatar: A clinico-epidemiological study

**DOI:** 10.5339/qmj.2026.7

**Published:** 2026-03-05

**Authors:** Wanis H. Ibrahim, Nuha Fituri, Nadin H. Abouzeid, Saif Addeen Khasawneh, Mohamed Elgara, Musa Hussein, Issam A. Bozom, Reyad Mohsen, Hafedh Ghazouani

**Affiliations:** 1 College of Medicine & Weill Cornell Medicine, Doha, Qatar; 2 Hamad General Hospital, Doha, Qatar; 3 National Centre of Cancer Care, Doha, Qatar; 4 Quality Management and Improvement Department, Hamad Medical Corporation, Doha, Qatar *Email: wanisian@yahoo.com

**Keywords:** Mesothelioma, epidemiology, pleura, asbestos, neoplasms

## Abstract

**Background:**

Malignant mesothelioma (MM) is a potentially fatal cancer that originates from the mesothelial surfaces, predominantly the pleura. Asbestos is the principal risk factor with a long latency period between exposure and disease development. The objective of this study was to investigate the clinical and epidemiological characteristics of MM in the State of Qatar.

**Methodology:**

This was a retrospective cohort study of all MM cases diagnosed in the State of Qatar during the period from January 1, 2015, to December 31, 2023. MM cases were identified from the Qatari National Cancer Registry Database and the Hamad Medical Corporation (HMC) Histopathology Database. Demographic, clinical, laboratory, radiologic, and survival data were collected from these two databases.

**Results:**

Among the total 22 MM cases diagnosed during the study period, migrant Egyptians constituted 15/22 (68.2%). The mean age for the study population was 57.9 ± 9.2 years with an age-standardized incidence rate of 24.1 cases per 1,000,000 individuals per year. The epithelioid histologic type was the predominant type (16/19; 84.2%), and most patients (13/14; 92.9%) were diagnosed at an advanced disease stage. The median overall survival (OS) was 16 months. The 1-year OS rate was 60.67%, dropping to 20.22% over 3 years. Though the differences were not statistically significant, patients with epithelioid histology and those with left-sided tumors experienced better OS outcomes.

**Conclusion:**

MM is predominantly a cancer of Egyptian migrants in the State of Qatar, with a comparable incidence to the international rates. Epithelioid histologic type is the most common type in the State of Qatar, and the majority of patients are diagnosed at an advanced disease stage. Despite a better OS rate compared to other countries, the prognosis of MM remains grave.

## 1. INTRODUCTION

Malignant mesothelioma (MM) is a fatal cancer with an insidious presentation that can arise from mesothelial cells of the pleura, the peritoneum, the pericardium, and the tunica vaginalis (membrane surrounding the testes) in men. While the pleura is the most common site, accounting for approximately 90% of cases, peritoneal involvement accounts for the remaining 10%.^1–3^ Both pericardial and tunica vaginalis MM account for less than 1% of MM.^2,4^ Asbestos exposure is the main risk factor for malignant pleural mesothelioma (MPM). Asbestos occurs in the soil and rocks as long fibers and has two main classes: the serpentines (chrysotile is the most common type) and the amphiboles (crocidolite, amosite, tremolite, anthophyllite, and actinolite are the main types).^5^ Recent studies have clearly linked all types of asbestos to pleural and peritoneal MM and to a lesser extent, lung cancer (unlike older studies that suggested a higher oncogenic role of amphibole asbestos).^5^ Recent research has also linked exposure to amphibole asbestos and autoimmune diseases such as systemic lupus erythematosus.^6^ The pathogenesis of MM results from the ability of asbestos fiber inhalation to induce repeated pleural inflammation, disruption of cellular mitosis, activation of proto-oncogenes, and the generation of free radicals. A prolonged latency period (15–60 years) from the time of asbestos exposure to the development of MM is usually needed.^7^ Other rarely reported risk factors for MM include ionizing radiation and genetic predispositions (germline mutations in the BRCA1-associated protein 1 [BAP 1] gene). The relationship between Simian virus 40 (SV40) and MM remains uncertain. While some studies have identified Simian virus 40 (SV40) nucleic acids in a large proportion of MM cases, more recent epidemiologic studies failed to confirm this observation.^8,9^ Although smoking is not directly linked to MPM, the combined smoking and asbestos exposure significantly increases the risk of lung cancer.^10,11^ Because of its carcinogenic risks, many environmental and cancer agencies, such as the “United States Environmental Protection Agency (US-EPA)” and the “International Agency for Research on Cancer (IARC)” have declared asbestos a proven human carcinogen.^12,13^ Over 60 countries in the world have banned the use of asbestos, and others have regulated its use. Nevertheless, many other countries, particularly developing ones with inadequate work safety regulations, are still using asbestos in many industries such as shipbuilding, mining, ceramics and cement manufacturing, automobile part production, especially brake lining, railroad repair, paper mill operations, and insulation work.^14^ Over the past few decades, the State of Qatar has witnessed a robust growth in its industrial sector with a huge investment in industrial companies that manufacture metal products, non-metallic mineral products, rubber and plastics, chemicals, and refined petroleum. Although the State of Qatar has banned the import and use of asbestos in 2010,^15^ the prolonged latency period of MM mandates the determination of its incidence, epidemiologic, and clinical characteristics in this country. To the best of our knowledge, the current study is the first to report such characteristics.

## 2. METHODS

This was a retrospective study of all MM cases diagnosed in the State of Qatar during the period from January 1, 2015, to December 31, 2023. Cases of MM diagnosed during the study period were identified from two primary sources: the Qatari National Cancer Registry Database of the National Center for Cancer Care and Research (NCCCR), and the Hamad Medical Corporation (HMC) Histopathology Database at Hamad General Hospital (HGH). All confirmed cases of MM diagnosed during the study period were included in the statistical analysis. Cases with lung cancer other than MM were excluded. For reliability and completeness of extracted data, we also performed an extensive search in patients’ electronic medical records, and investigators received training on how to complete the data sheet. Two senior investigators independently reviewed the data collected by other investigators. For mortality and survival outcomes, patients were censored until any of the following happened: (a) the patient died, (b) the patient had not yet died by the time the study was closed, or (c) the time of the last follow-up if a patient was lost to follow-up. Qualitative and quantitative data were expressed as the frequency with percentage and mean ± standard deviation (SD), with median and interquartile range (IQR). The demographic and clinical characteristics of the study population were summarized using descriptive statistics. The chi-squared test or Fisher’s exact test (for small cell frequencies) was used to describe the associations between qualitative or categorical variables when appropriate. Incidence rates were calculated as the number of new cases per 1,000,000 person-years with 95% confidence intervals (CIs), and the World Health Organization “World Standard Population 2000–2025” was utilized for age standardization.^16^ The probability of survival was determined by the appropriate survival tables and Kaplan-Meier curves. The log-rank test was used to compare the survival distributions of different groups. Statistical significance was determined by a *P*-value of less than 0.05. Statistical analyses were performed using StataCorp (Stata Corp., 2015; Stata Statistical Software: Release 14, StataCorp LP, College Station, TX). The study was approved by the Medical Research Center of HMC (proposal ID: MRC-01-24-002; approval date: March 5, 2024).

## 3. RESULTS

**Figure 1.**
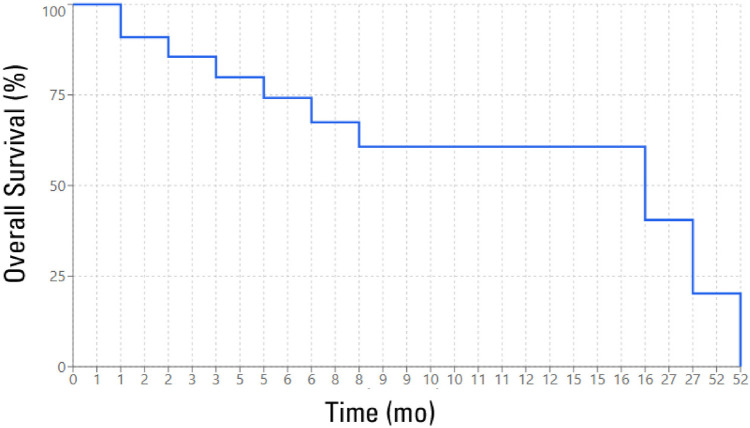
Survival analysis. The Kaplan-Meier curve.

A total of 22 MM (all MPM) cases were diagnosed during the study period. The demographic, clinical, laboratory, and radiological characteristics are shown in Tables 1 and 2. The mean and median ages for the study population were 57.9 ± 9.2 and 60 (IQR: 54.0–64.3) years, respectively. The age-standardized incidence rate of MM in the State of Qatar was 24.1 cases per 1,000,000 individuals per year. Males constituted most patients (90.9%). Interestingly, most patients (68.2%) were Egyptian migrants, resident in Qatar. Smoking history was documented in 70% of patients. Chest pain and breathlessness were the most common reported symptoms, with a mean duration of 34 days. Most patients had the epithelioid histologic type of mesothelioma (84.2%) and were diagnosed at advanced disease stage (92.9% with stage IV). The most common radiologic finding was a pleural effusion (100%), and video-assisted thoracoscopic surgery (VATS) was the method used to confirm the diagnosis in approximately 62% of cases. The median OS was 16 months, with a mean survival of 9.14 months. The 1-year OS rate was 60.67%, dropping to 20.22% over 3 years (Figure 1). Patients with epithelioid histology (mean survival, 10.38 months) and those with left-sided tumors (mean survival, 12.88 months) experienced better outcomes than patients with non-epithelioid subtypes (mean survival, 5.83 months) and those with right-sided tumors (mean survival, 7.0 months). However, the differences were not statistically significant (Table 3).

## 4. DISCUSSION

The most striking finding in the current study is the high percentage of Egyptian migrants affected by MM (68.2% of patients). Qatar relies heavily on a migrant workforce (particularly men) for its industrial development. Arabs, including Egyptians, represent the second-largest workforce after South Asians.^17^ Asbestos use in Egypt has been recognized since the times of the pharaohs, when it was used for mummification. However, the recent asbestos industry was initiated in 1948 by Sigwart El–Maasara Company in South Cairo. By the year 2004, 14 asbestos factories were established in Egypt. Some places in Egypt with high asbestos risks include Shobra Elkhema, El-Maasara, Helwan, and El Zytoon. In 2004, the ministerial council in Egypt decided to ban asbestos imports, and Siegwart plants were closed in the same year.^18,19^ Nevertheless, because of the prolonged latency period of MM, the risk of developing MM will remain for a considerable period of time. It has been estimated that the incidence of MM in Egypt will reach its peak around 2040.^18–21^ The age-standardized incidence rate of MM in the State of Qatar was 24.1 cases per 1,000,000 individuals per year. Such an incidence is comparable to the international figures. Data from European countries revealed an average incidence of 20 cases per million inhabitants per year.^22^ Nevertheless, MM incidence varies worldwide, with the lowest reported from Japan (7 per million) and the highest from Australia (40 per million).^23–26^ In some industrialized European countries, the incidence has steadily increased over the last 20 years and is expected to peak by 2020 to 2025.^22,24^ In other countries (such as the United Kingdom and Sweden) where asbestos control measures were implemented during the 1970s, the incidence rates are declining.^22,25,27^ Despite the confirmation of grave MM prognosis in the current study, we found a better median OS (16 months) in Qatar compared to other countries. In the most recent and largest National Cancer Database (NCDB) of the United States, released in August 2022, Bou-Samra et al. found a median OS of 10.3 months among patients with MPM.^28^ The median OS rates reported from Egypt, the Netherlands, and the United Kingdom were 9, 9.3, and 11.5 months, respectively.^29–31^ The better survival rates observed in the State of Qatar can be attributed to several factors, including the availability of free-of-charge advanced treatment options to all cancer patients living in the country, the timely access to oncology and multidisciplinary care, and the utilization of state-of-the-art testing for oncology cases.^32^ Nevertheless, this finding should be interpreted with caution due to the small sample size. The two strongest prognostic factors for mesothelioma reported in the literature are the stage of the disease and the histologic type. Sarcomatoid and biphasic histologic subtypes have the worst outcomes, while epithelioid mesothelioma has the best outcome. Older age and poor performance status also exhibit a worse prognosis.^33^ In the current study, patients with epithelioid histology (mean survival, 10.38 months) and those with left-sided tumors (mean survival, 12.88 months) experienced better outcomes than patients with non-epithelioid subtypes (mean survival, 5.83 months) and those with right-sided tumors (mean survival, 7.0 months). However, the differences were not statistically significant. The small sample size in this study has also influenced the statistical significance of various prognostic factors, including age, histologic subtypes, and smoking status. Our study is the first to describe the epidemiological and clinical characteristics of MM in the State of Qatar. It has also determined the population at risk of developing this lethal cancer and hence can help healthcare policymakers in the country to initiate an MM screening program. Another strength of this study is the extensive search in the patient’s medical records, along with both pathology and the national cancer registry, to ensure accuracy and consistency of the collected data. In the State of Qatar, all cancer cases, including MM, are referred to and treated in a single cancer care center (NCCCR), and all cancer pathological specimens are examined in the central laboratory of HMC. As MM cases were obtained from registries of these two facilities, we are confident that the results of our study can be generalized to the whole country.

### 4.1 Study limitations

An important limitation of the current study is its retrospective nature (missing data) and the small sample size, which did not permit an extensive study of the risk factors, details of asbestos exposure, and the treatment effects on the outcomes.

## 5. CONCLUSION

MM is predominantly a cancer of Egyptian migrants in the State of Qatar, with a comparable incidence to the international rates. Epithelioid histologic type is the most common type in the State of Qatar, and the majority of patients are diagnosed at an advanced disease stage. Despite a better OS rate compared to other countries, the prognosis of MM remains grave. Better, but statistically nonsignificant, survival outcomes have been observed with epithelioid histologic type and left-sided tumors.

## AUTHOR’S CONTRIBUTION

WI: Conceptualization, Methodology, Investigation & Analysis, Visualization, Writing – Original Draft, Resources, Supervision, Project Administration. NF: Methodology, Data Curation, Software & Validation, Investigation & Analysis, Visualization, Project Administration. NA: Methodology, Data Curation, Software & Validation, Investigation & Analysis, Visualization, Project Administration. SK: Methodology, Data Curation, Software & Validation, Investigation & Analysis, Visualization, Project Administration. ME: Methodology, Data Curation, writing – Original Draft, Writing – Review & Editing. MH: Methodology, Data Curation, writing – Original Draft, Writing – Review & Editing. IB: Conceptualization, Methodology, Data Curation, writing – Original Draft, Writing – Review & Editing. RM: Conceptualization, Methodology, Data Curation, writing – Original Draft, Writing – Review & Editing. HG: Methodology, Software & Validation, Investigation & Analysis.

## CONFLICT OF INTEREST

The authors declared no potential conflicts of interest.

## IRB APPROVAL

The study was approved by the Medical Research Centre of Hamad Medical Corporation (Proposal ID: MRC-01-24-002).

## Figures and Tables

**Table 1.** Demographic characteristics of the study population.

	No. (%)
Gender (*N* = 22)	
Males	20 (90.9%)
Females	2 (9.1%)
Age (*N* = 22)	
Age (years)	Mean: 57.9 ± 9.2 Median (IQR): 60.0 (54.0–64.3)
Nationality (*N* = 22)	
Bangladeshi	2 (9.1%)
Egyptian	15 (68.2%)
Jordanian	1 (4.5%)
Kenyan	1 (4.5%)
Pakistani	1 (4.5%)
Qatari	1 (4.5%)
Sudanese	1 (4.5%)
Smoking history (*N* = 20)	
Smokers	14 (70.0%)
Non-smokers	6 (30%)
Mean pack year	35.9±27.9
Occupation (*N* = 9)	
Accountant	1 (11.1%)
Asbestos cement manufacturing	1 (11.1%)
Desk job	1 (11.1%)
Driver	1 (11.1%)
Asbestos	1 (11.1%)
Lawyer	1 (11.1%)
Ship building	1 (11.1%)
Railroad repair	1 (11.1%)
Soldier	1 (11.1%)

**Table 2.** Clinical, laboratory, and radiologic characteristics of the study population.

Presenting symptom(s) (*N* = 22)	
Cough	3 (13.6%)
Chest pain	7 (31.8%)
Breathlessness	5 (22.7%)
Multiple respiratory symptoms	7 (31.8%)
**Duration of symptoms (days)**	
Mean	34 ± 69
Median	10.0
Minimum	1
Maximum	300
**Disease site (*N* = 22)**	
Right	13 (59.1%)
Left	9 (40.9%)
**Histologic type (*N* = 19)**	
Epithelioid	16 (84.2%)
Unspecified	3 (15.8%)
**Stage at time of diagnosis (*N* = 14)**	
Stage 1A	1 (7.1%)
Stage IV	13 (92.9%)
**Pleural effusion type (*N* = 19)**	
Lymphocytic	19 (100%)
**Method of diagnosis (*N* = 21)**	
Medical thoracoscopy	8 (38.1%)
VATS	13 (61.9%)
**Radiologic findings (*N* = 22)**	
Pleural effusion	22 (100%)
Pleural plaques	1 (4.5%)
Pleural thickening	5 (22.7%)
Lung fibrosis	1 (4.5%)
Lung collapse/consolidation	5 (22.7%)
Mediastinal lymphadenopathy	3 (13.6%)
Palpable chest wall mass	1 (4.5%)

VATS: Video-assisted thoracoscopic surgery.

**Table 3.** Log-rank test for survival differences between subgroups.

Variable	Group 1				Group 2				Statistical test	
	Category	*N*	Events	Mean OS (months)	Category	*N*	Events	Mean OS (months)	*P* value	Sig.
Age	Age ≤60	11	5	6.36	Age >60	11	5	11.91	**0.397**	No
Nationality	Egyptian	15	6	9.93	Other	7	4	7.43	**0.312**	No
Histology	Epithelioid	16	8	10.38	Non-epithelioid	6	2	5.83	**0.809**	No
Smoking	Smoker	15	9	9.53	Non-smoker	7	1	8.29	**0.184**	No
Tumor site	Right lung	14	6	7	Left lung	8	4	12.88	**0.473**	No
